# Three *Starch Synthase IIa* (*SSIIa*) Alleles Reveal the Effect of SSIIa on the Thermal and Rheological Properties, Viscoelasticity, and Eating Quality of Glutinous Rice

**DOI:** 10.3390/ijms24043726

**Published:** 2023-02-13

**Authors:** Tsukine Nakano, Naoko Crofts, Satoko Miura, Naoko F. Oitome, Yuko Hosaka, Kyoko Ishikawa, Naoko Fujita

**Affiliations:** 1 Department of Biological Production, Faculty of Bioresource Sciences, Akita Campus, Akita Prefectural University, 241-438 Kaidobata-Nishi, Shimoshinjo-Nakano, Akita 010-0195, Japan; 2 Department of Biotechnology, Faculty of Bioresource Sciences, Akita Campus, Akita Prefectural University, 241-438 Kaidobata-Nishi, Shimoshinjo-Nakano, Akita 010-0195, Japan

**Keywords:** glutinous rice, starch, *starch synthase IIa*, low gelatinization temperature, texture

## Abstract

Glutinous rice accumulates amylose-free starch and is utilized for rice cakes and crackers, owing to the loss of the *Waxy* gene which encodes granule-bound starch synthase I (GBSSI). *Starch synthase IIa (SSIIa)* elongates amylopectin chains with a degree of polymerization (DP) of 6–12 to 13–24 and greatly influences starch properties. To elucidate the relationship between the branch length of amylopectin and the thermal and rheological properties, viscoelasticity, and eating quality of glutinous rice, three allelic near isogenic lines with high, low, or no SSIIa activity were generated (designated as *SS2a wx*, *ss2a^L^ wx*, and *ss2a wx*, respectively). Chain length distribution analyses revealed that *ss2a wx* exhibited the highest short chain (DP < 12) number and lowest gelatinization temperature, whereas *SS2a wx* showed the opposite results. Gel filtration chromatography showed that the three lines contained essentially no amylose. Viscoelasticity analyses of rice cakes stored at low temperature for different durations revealed that *ss2a wx* maintained softness and elasticity for up to 6 days, while *SS2a wx* hardened within 6 h. Sensory evaluation was consistent with mechanical evaluation. The relationship of amylopectin structure with the thermal and rheological properties, viscoelasticity, and eating quality of glutinous rice is discussed.

## 1. Introduction

Glutinous rice has an attractive sticky texture and is used for preparing desserts and snacks such as rice cakes and crackers, thus contributing to the food culture of Asian countries. The starch in glutinous rice is composed of α-1,6-branched glucose polymers of amylopectin and lacks α-1,4-linked linear glucose polymers of amylose [1,2,3]. Amylose in rice endosperm is exclusively synthesized by granule-bound starch synthase I (GBSSI) encoded by the *Waxy* (or *GBSSI*) gene, while the amylose in vegetative tissues is synthesized by GBSSII [4,5]. GBSSs are targeted to starch granules via noncatalytic proteins harboring coiled-coil domains and carbohydrate-binding module 48, named as GBSS-binding protein (OsGBP), a homolog of the protein targeted to starch (PTST) originally identified in Arabidopsis [6,7]. The abundance of GBSSI directly correlates with the apparent amylose content and texture of cooked rice [1,2,7]. Starch in non-glutinous rice generally contains 15–30% amylose, depending on the amount of GBSSI; the lower the amylose content, the stickier the texture of cooked rice [1,2,3,8,9]. Loss of GBSSI and/or OsGBP leads to almost no amylose accumulation and consequently glutinous rice [1,3,7,9]. A glutinous japonica rice mutant, EM21, was isolated from the N-methyl-N-nitrosourea (MNU) treatment-derived mutant population of Kinmaze [10]. A single nucleotide polymorphism (SNP) in the seventh exon of GBSSI resulted in a premature stop codon (replacing tryptophan at amino acid position 235) [11], leading to no GBSSI protein and consequently a negligible amount of amylose in the endosperm [3,12]. Therefore, variation in rice starch properties is attributed to amylopectin.

Amylopectin is synthesized through the finely balanced synergistic actions of starch synthases (SSs, which elongate glucan chains), starch branching enzymes (BEs, which generate branches), and starch debranching enzymes (DBEs, which remove branches); these are mainly isoamylase (ISA1), and there is a minor contribution of pullulanase (PUL) [13,14]. These enzymes physically interact with each other to form multiprotein complexes [15]. Starch biosynthetic enzymes in each class exist as multiple isoforms, and exhibit different spatiotemporal expression patterns and preferred glucan primer and end product structures [13,16,17]. For instance, in rice endosperm, SSIIIa elongates long backbone glucan chains with a degree of polymerization (DP) > 30 that connect amylopectin clusters, while SSI elongates short chains (DP 6 and 7) to DP 8–12, which are further elongated (DP 13–24) by SSIIa (also known as ALK or SSII-3), depending on the cultivar [14]. Since the typical indica rice cultivars possess the *SS2a* allele (also known as *SSIIa^I^* or *ALK^c^*), which encodes the highly active SSIIa protein, these cultivars contain more intermediate amylopectin chains with DP 13–24, displaying L-type amylopectin structure, and exhibit a higher gelatinization temperature than the typical japonica rice cultivars (*ss2a^L^*, also known as *SSIIa^J^* or *ALK^a/b^*), which contain S-type amylopectin [3,18,19,20]. SSIIa in EM204, a non-glutinous *ss2a* null mutant of japonica rice isolated from the MNU-mutagenized population of Kinmaze, theoretically lacks 15 amino acids, corresponding to the sixth exon of SSIIa, owing to mutation at the last nucleotide of the fifth intron; however, it actually has almost no SSIIa protein [21]. The loss of SSIIa resulted in even less intermediate amylopectin chains (DP 13–24) and more short amylopectin chains (DP < 12), leading to a considerably lower gelatinization temperature compared with its parental japonica rice [21]. Hence, typical japonica rice cultivars possess less, but some, SSIIa activity compared with indica rice [19,21].

Glutinous rice cultivars have undergone the domestication process and are suitable for the preparation of different end products [22,23]. Considerable differences in starch structure and gelatinization, retrogradation, rheological, and pasting properties have been observed among these cultivars [12,24,25]. However, genetic factors responsible for the differences in the properties of each cultivar remain largely unknown. Recent analyses of nucleotide polymorphisms in the genes encoding starch biosynthetic enzymes suggest that SSs, BEs, and PUL influence the thermal and/or pasting properties of glutinous rice cultivars, and SSIIa seems to have the greatest effect [26]. For example, Kantomochi 172 possesses high activity-type SSIIa and produces less short amylopectin chains (DP 6–12) and more long amylopectin chains (DP 13–24), resembling the typical L-type amylopectin structure of its parental indica rice, IRAT 109 [27,28,29]. The rice cakes prepared from Kantomochi 172 rapidly harden and are less elastic and less adhesive, giving this cultivar suitable properties for preparing rice cracker dough [29]. By contrast, Aichimochi 126 contains fewer long amylopectin chains and more short amylopectin chains because of the loss of BEI, thus gelatinizing at a lower temperature, exhibiting slow retrogradation, and producing softer rice cake dough, which is suitable for preparing dumplings [30,31]. Therefore, amylopectin branch length is a crucial determinant of glutinous rice properties.

Recently, we demonstrated that the heading date and/or seed developing temperature affect the chain length distribution and gelatinization temperature of starch in non-glutinous rice lines lacking SSIIa [32]. In addition, indica and japonica rice cultivars exhibit a few nucleotide polymorphisms [33,34]. Furthermore, combinations of the absence and presence of multiple starch biosynthetic enzymes in non-glutinous rice have been shown to alter amylopectin branch structure, resulting in altered thermal and digestive properties of rice [17,35,36].

Taking all of the above findings into account, we speculated that *ss2a* null mutants of glutinous rice would exhibit a further reduction in gelatinization temperature, thus enabling the production of even softer rice cakes. In addition, comparisons among *SS2a*, *ss2a^L^*, and *ss2a* alleles in the *wx* background would reveal, in detail, the effect of SSIIa on amylopectin branch length and its relationship with the thermal and rheological properties, viscoelasticity, and eating quality of glutinous rice. In the present study, we aimed to evaluate the precise effects of high, low, and no SSIIa activity on glutinous rice properties using near isogenic rice lines (NILs) to control for the flowering time and the allelic status of non-target starch biosynthetic genes. Three NILs (*SS2a wx* (or *gbss1*), *ss2a^L^ wx*, and *ss2a wx*) were generated by backcrossing with an elite rice cultivar Akita 63, which has early flowering and high yielding traits (Akita 63 [37]), so these lines could be used for practical applications in the food industry in the near future. The starch structure, thermal and rheological properties, viscoelasticity, and eating quality of glutinous rice were investigated.

## 2. Results

### 2.1. Genotype of Glutinous Rice NILs with Different SSIIa Alleles

**Figure 1:**
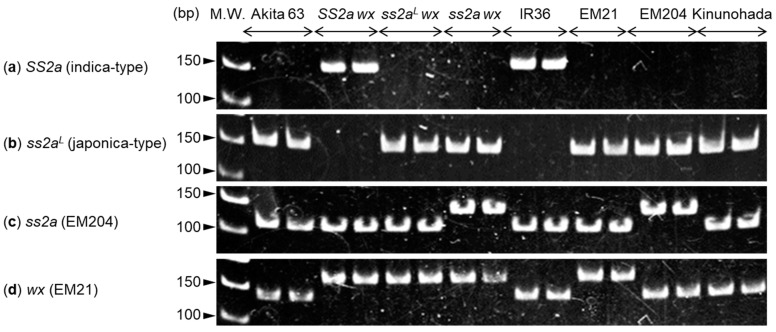
Analysis of nucleotide polymorphisms and mutation sites in *starch synthase* (*SS*) *IIa* and *waxy* (*Wx*) genes. (**a**,**b**) Genotyping the high activity-type *SS2a* allele derived from indica rice (**a**) and low activity-type *ss2a^L^* allele derived from japonica rice (**b**) based on nucleotide polymorphisms in the first exon of the *SSIIa* gene. (**c**) Identification of the *ss2a* allele derived from EM204 based on the mutation site in the fifth intron of the *SSIIa* gene. (**d**) Identification of the *wx* allele derived from EM21 based on the mutation site in the seventh exon of the *Wx* gene. Two samples each were prepared from individual seedlings of the same genotype.

Nucleotide polymorphisms and mutation sites in *SSIIa* and *wx* genes were analyzed using genomic DNA isolated from Akita 63, *SS2a wx*, *ss2a^L^ wx*, *ss2a wx*, IR36, EM21, EM204, and Kinunohada (Figure 1a–d). *SS2a* and *ss2a^L^* alleles were distinguished on the basis of nucleotide polymorphisms found in the first exon using established allele-specific markers [38]. The results showed that the *SS2a* allele-specific PCR product (160 bp) was generated in *SS2a wx* and IR36 (Figure 1a), while the *ss2a^L^* allele-specific PCR product (160 bp) was generated in Akita 63, *ss2a^L^ wx*, *ss2a wx*, EM21, EM204, and Kinunohada (Figure 1b). Since the *SSIIa* gene in *ss2a wx* and EM204 was originally derived from the japonica rice cultivar Kinmaze [21], the nucleotide polymorphism in the first exon of *SSIIa* was the same as that in Akita 63, *ss2a^L^ wx*, EM21, and Kinunohada. The *ss2a* allele was identified on the basis of a mutation in the fifth intron using a derived cleaved amplified polymorphic sequence (dCAPS) marker [21]; the 141 bp PCR product was not cleaved by *Nla*IV in *ss2a wx* and EM204 but was digested into 111 bp and 30 bp fragments in the other lines, although the 30 bp fragment was too small to detect. Similarly, the *wx* allele was identified using a dCAPS marker based on the mutation in the seventh exon; the 167 bp PCR product was not cleaved by *Bse*NI in *SS2a wx*, *ss2a^L^ wx*, *ss2a wx*, and EM21 but was digested into 135 bp and 32 bp fragments in the other lines, although the 32 bp fragment was too small to detect. Despite being a glutinous rice cultivar, the mutation site in the *wx* gene of Kinunohada was different from that in the *wx* allele of *SS2a wx*, *ss2a^L^ wx*, *ss2a wx*, and EM21. The *SSIIa* and *wx* genotypes of rice lines used in this study are summarized in Table 1. IR36 is known to possess the *Wx^a^* (or *GBSS1*) allele [3], whereas Akita 63 and Kinmaze, the parental line of EM204, are known to possess the *Wx^b^* (or *gbss1^L^*) genotype [3,39]; the *Wx^a^* and *Wx^b^* alleles can be distinguished from each other based on nucleotide polymorphisms in the first intron of the *Wx* gene [40]. These results show that rice lines generated in this study possess the expected genotypes.

### 2.2. Expression and Starch Granule Affinity Analyses of SSI, SSIIa, and GBSSI Proteins

**Figure 2:**
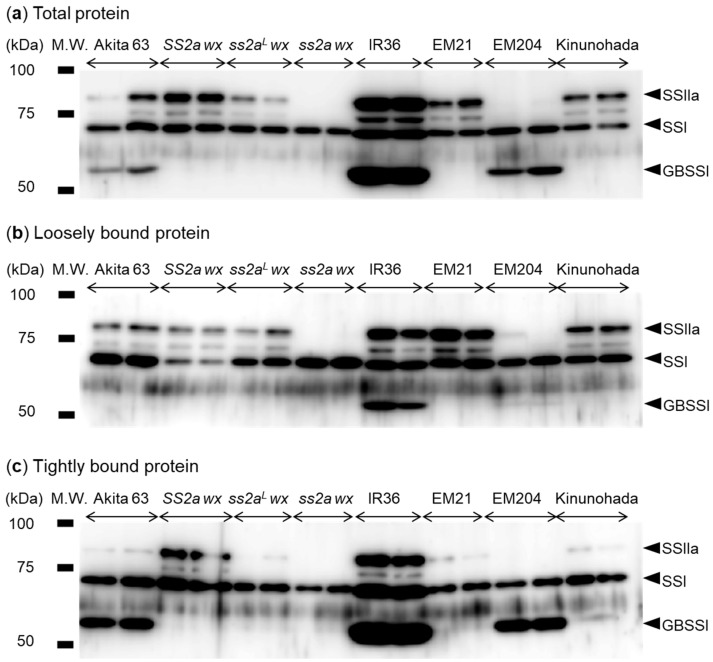
Analysis of SSI, SSIIa, and granule-bound starch synthase (GBSS) I protein abundance in mature seeds. (**a**) Analysis of total protein extracted from mature seeds by Western blotting using anti-SSI, -SSIIa, and -GBSSI antibodies. SSI served as a loading control. (**b**,**c**) Western blotting analysis of proteins loosely bound (**b**) and tightly bound (**c**) to starch granules. Two samples each were prepared from individual grains of the same genotype.

Expression levels of SSIIa and GBSSI were determined by Western blotting. Total protein was extracted from mature seeds and probed with the mixture of isoform-specific antibodies directed against SSI, SSIIa, or GBSSI (Figure 2a). SSI (loading control) was detected in all rice lines (Figure 2a). Faint signals between the SSI and SSIIa bands, representing degradation products of SSIIa, were observed in all rice lines except *ss2a wx* and EM204 (Figure 2). Although total protein was extracted using an equal ratio of rice powder and extraction buffer for all lines, higher levels of SSI, SSIIa, and GBSSI were detected in IR36. The SSIIa protein was completely absent in *ss2a wx* and EM204. The amount of SSIIa was the highest in IR36, considerably high in *SS2a wx*, and lower in Akita 63, *ss2a^L^ wx*, EM21, and Kinunohada (Figure 2a). The GBSSI protein was not detected in *SS2a wx*, *ss2a^L^ wx*, *ss2a wx*, EM21, and Kinunohada, confirming their *wx* genotype (Figure 2a). The level of GBSSI was low in Akita 63, slightly higher in EM204, and highest in IR36 (Figure 2a), which is consistent with previous studies [3,21].

Since SSIIa from indica rice tends to associate with starch granules, unlike that of japonica rice [3], the starch granule affinity of SSI, SSIIa, and GBSSI was investigated in this study (Figure 2b,c). A large proportion of SSIIa was associated with starch granules in *SS2a* lines, such as IR 36 and *SS2a wx*, but less SSIIa was associated in *ss2a^L^* lines, including Akita 63, *ss2a^L^ wx*, EM21, and Kinunohada (Figure 2b,c). This suggests that the four known amino acid substitutions between indica and japonica SSIIa proteins [19] affect their affinity for starch granules. SSIIa was not detected in either the loosely bound or tightly bound protein fractions of *ss2a wx* and EM204 (Figure 2b,c), as expected.

### 2.3. Activities of SSIIa and Other Starch Biosynthetic Enzymes

**Figure 3:**
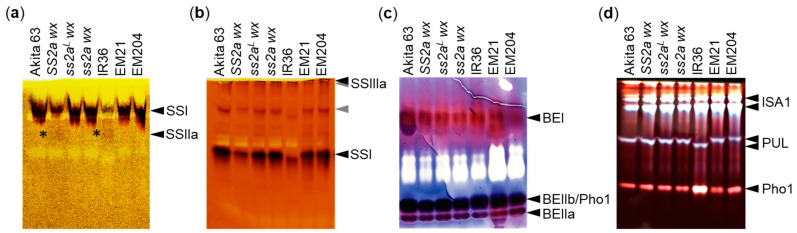
Activities of SSIIa and other starch biosynthetic enzymes visualized by zymogram analysis. (**a**) SSIIa activity (indicated by asterisks) visualized by polyacrylamide gel electrophoresis (PAGE) using a native gel containing maize amylopectin as a primer in the presence of ADP-glucose. (**b**) SSI and SSIIIa activities visualized by PAGE using a native gel containing oyster glycogen as a primer in the presence of ADP-glucose. Gray arrowheads likely represent the transferase or hydrolase activity of enzymes, as they also appeared in the absence of the substrate (Figure S1b). (**c**) Branching enzyme (BE) I, BEIIa, and BEIIb activities visualized by phosphorylase stimulation assay. (**d**) Activities of debranching enzymes (DBEs; ISA1 and PUL) and Pho1 visualized by PAGE using a native gel containing potato amylopectin. All gels were stained with iodine after the reaction.

Since the SSIIa protein was detected in SS2a and ss2a^L^ lines, its activity was compared between the two genotypes by zymogram analysis (Figure 3a). Soluble proteins were extracted from seeds at the mid-developmental stage, and SSIIa activity was examined by polyacrylamide gel electrophoresis (PAGE) using native gel containing maize amylopectin in the presence (Figure 3a) or absence (Figure S1a) of ADP-glucose (substrate). Faint SSIIa activity was observed in SS2a wx and IR36 (SS2a allele); however, no SSIIa activity was detected in Akita 63, ss2a^L^ wx, and EM21 (ss2a^L^ allele), even though the SSIIa protein was expressed in these lines (Figure 2). The ss2a wx and EM204 lines showed no SSIIa activity, as expected. Thus, the results of Western blotting analysis of SSIIa and GBSSI (Figure 2) and zymogram analysis of SSIIa (Figure 3a) endorsed that the three NILs generated in this study possess the expected genotypes.

SSI and SSIIIa activities were visualized by native-PAGE using oyster glycogen in the presence (Figure 3b) or absence (Figure S1b) of ADP-glucose (substrate). Compared with SS2a lines, SSI activity appeared to be stronger in ss2a^L^ and ss2a lines, such as Akita 63, ss2a^L^ wx, ss2a wx, EM21, and EM204; this may be because the amount of SSI in the soluble protein fraction in these lines was higher, as the amount of SSI tightly associated with starch granules was lower than those of the lines with SS2a (Figure 2c). The activity of SSIIIa was similar among all lines. Faint bands, indicated by gray arrowheads and likely representing transferase or hydrolase activities, also appeared in the absence of the substrate (Figure S1b). The activities of other starch biosynthetic enzymes, such as BEs, ISA1, and PUL, were also similar across most of the lines, although the mobility of PUL was faster in IR36 than in other lines. Additionally, the activity of phosphorylase 1 (Pho1) was stronger in IR36 than in other lines (Figure 3c,d).

### 2.4. Seed Morphology and Seed Weight

**Figure 4:**
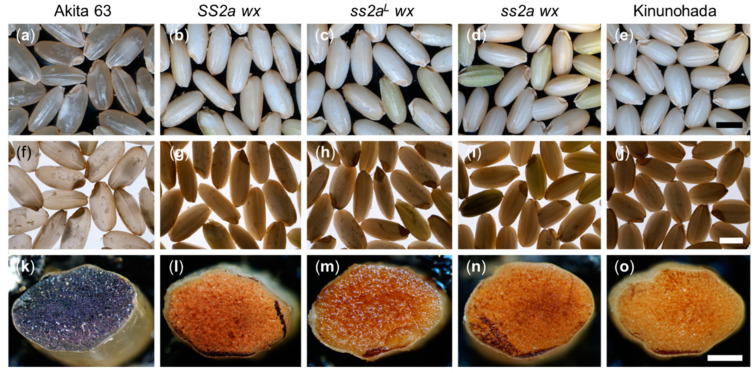
Morphology of rice grains and iodine staining of seed cross-sections. (**a**–**j**) Analysis of the morphology of dehulled rice seeds by stereomicroscopy with illumination from above (**a**–**e**) and below (**f**–**j**). Scale bars, 3 mm. (**k**–**o**) Cross-sections of rice seeds stained with iodine and observed under a stereomicroscope. Scale bars, 1 mm.

Morphologies of dehulled rice seeds were observed using a stereomicroscope with light from above (Figure 4a–e) and below (Figure 4f–j). The SS2a wx, ss2a^L^ wx, ss2a wx, and Kinunohada seeds were white and opaque, which is typical of glutinous rice seeds, when illuminated from above (Figure 4g–j). This is consistent with the appearance of other wx rice seeds [41]. The seeds of non-glutinous rice Akita 63 were translucent when illuminated from above (Figure 4f). When illuminated from below, the seeds of glutinous rice lines SS2a wx, ss2a^L^ wx, ss2a wx, and Kinunohada appeared dark (Figure 4g–j).

To visualize the presence or absence of amylose, cross-sections of dehulled rice seeds were stained by iodine solution and observed under a stereomicroscope. Seeds of the non-glutinous rice line Akita 63 stained dark purple, indicating the presence of amylose (Figure 4k). On the contrary, seed cross-sections of glutinous rice lines SS2a wx, ss2a^L^ wx, ss2a wx, and Kinunohada stained brown, indicating the absence of amylose (Figure 4l–o).

Next, the weight of dehulled grains was measured, and the average weight of 20 grains was calculated for each line. The per-grain weight of SS2a wx, ss2a^L^ wx, and ss2a wx lines was approximately 27.8, 27.1, and 27.5 mg, respectively (Table 2), which was significantly (115%) greater than that of the commonly consumed glutinous rice cultivar Kinunohada (23.6 mg) and the parental glutinous rice line EM21 (17.6 mg), indicating that the SS2a wx, ss2a^L^ wx, and ss2a wx lines could be used for commercial purposes.

### 2.5. Amylose Content and Amylopectin Structure

Iodine staining indicated that the SS2a wx, ss2a^L^ wx, ss2a wx, and Kinunohada seeds were amylose-free and glutinous (Figure 4). To determine the precise apparent amylose content of these lines, starch was purified from mature seeds, debranched by isoamylase, and separated by gel filtration chromatography using a series of single HW-55S and triple HW-50S Toyopearl columns (Table 2, Figure S2). Fractions I, II, and III of starch contain amylose, long amylopectin chains, and short amylopectin chains, respectively (Figure S2). The apparent amylose content of the japonica rice cultivar Akita 63 was approximately 17%, while those of SS2a wx, ss2a^L^ wx, ss2a wx, EM21, and Kinunohada were negligible (0.5%, 1.0%, 2.1%, 0.5%, and 1.0%, respectively) (Table 2). The amount of amylose in IR36, EM21, and EM204 was previously determined to be 26–27% [3,21], 0.5% [3], and 24% [21], respectively. Although GBSSI was not detected in SS2a wx, ss2a^L^ wx, and ss2a wx (Figure 2), a very small amount of glucan was detected in fraction I, which could be amylose synthesized by GBSSII. Indeed, the iodine-stained endosperm cross-section showed a slightly dark brown area at the periphery of SS2a wx, ss2a^L^ wx, and ss2a wx grains (Figure 4l–o). The profile of fraction III varied depending on the SSIIa allele (Figure S2). SS2a wx showed a single peak (Figure S2c), while Akita 63, ss2a^L^ wx, ss2a wx, and Kinunohada showed a small trough at the top of fraction III (Figure S2a,b,d,e), which was deeper in ss2a wx (Figure S2e) than in Akita 63, ss2a^L^ wx, and Kinunohada (Figure S2a,b,e). These findings indicate that short amylopectin chain length distribution varies among rice lines, depending on the SSIIa allele.

**Figure 5:**
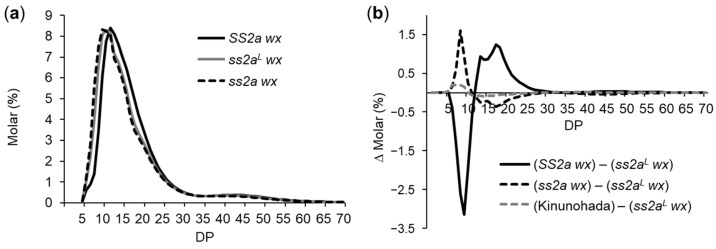
Analysis of amylopectin branch structure of endosperm starch by capillary electrophoresis. (**a**) Chain length distribution pattern of SS2a wx (black solid line), ss2a^L^ wx (gray solid line), and ss2a wx (black dotted line). (**b**) Subtraction curves showing the detailed effects of SSIIa alleles on amylopectin branch structure in SS2a wx (black solid line), ss2a wx (black dotted line), and Kinunohada (gray dotted line) compared with ss2a^L^ wx. Each panel is one typical representative dataset of at least three replicates.

Since amylose content reflects the Wx genotype and GBSSI content, and the SS2a wx, ss2a^L^ wx, ss2a wx, and Kinunohada lines were found to be glutinous with some variation in amylopectin structure, their amylopectin structure was determined by capillary electrophoresis to reveal the effect of SSIIa alleles (Figure 5a,b). Analysis of the chain length distribution patterns of SS2a wx, ss2a^L^ wx, and ss2a wx indicated that the amylopectin chains of ss2a wx were slightly shorter than those of ss2a^L^ wx, while SS2a wx possessed slightly longer amylopectin chains than ss2a^L^ wx (Figure 5a).

To precisely evaluate the effect of SSIIa on amylopectin branch length, subtraction curves were generated (Figure 5b). The values of the chain length distribution pattern in ss2a^L^ wx were subtracted from those in SS2a wx, ss2a wx, or Kinunohada (Figure 5b). A subtraction curve clearly showed the differences seen in Figure 5a. The ss2a wx line contained more short amylopectin chains (DP 5–12) and fewer long amylopectin chains (DP 13–24) than ss2a^L^ wx, while SS2a wx contained fewer short amylopectin chains (DP 5–12) and more long amylopectin chains (DP 13–30). Chain length distribution in Kinunohada was essentially the same as that in ss2a^L^ wx, except for a very slight increase in the number of short chains (DP 5–12) in Kinunohada (Figure 5b). These results suggest that amylopectin branch structure is correlated with the strength of SSIIa activity; the lower the SSIIa activity, the higher the number of short amylopectin chains and the lower the number of long amylopectin chains, and vice versa. These results are in agreement with the function of SSIIa [18,19,21].

### 2.6. Thermal and Pasting Properties of Endosperm Starch

Amylopectin branch length greatly affects the thermal and pasting properties of starch [20,42,43,44]. Therefore, gelatinization temperature was measured by differential scanning calorimetry (DSC) using starch purified from mature seeds (Table 3; Figure S3). The onset temperature of SS2a wx, ss2a^L^ wx, and ss2a wx was approximately 67 °C, 58 °C, and 50 °C, respectively; the peak gelatinization temperature of these lines was approximately 73 °C, 65 °C, and 61 °C, respectively (Table 3; Figure S3); and the conclusion temperature of these lines was 83 °C, 75 °C, and 72 °C, respectively. The gelatinization enthalpy of SS2a wx, ss2a^L^ wx, and ss2a wx was approximately 19, 17, and 14 J/g, respectively. Peak gelatinization temperature was closely correlated with SSIIa activity and amylopectin branch structure; the lower the SSIIa activity, the higher the number of short amylopectin chains and the lower the gelatinization temperature (Table 3; Figure S3); on the contrary, the higher the SSIIa activity, the higher the number of long amylopectin chains and the higher the gelatinization temperature (Table 3; Figure S3). In addition, the lower the SSIIa activity, the lower the amount of heat energy required to gelatinize the starch.

The peak gelatinization temperature of Kinunohada (approximately 62 °C) was lower than that of ss2a^L^ wx (approximately 65 °C) but slightly higher than that of ss2a wx (approximately 61 °C). Although Kinunohada also likely possesses the ss2a^L^ allele, analysis of chain length distribution pattern revealed a slightly higher number of short amylopectin chains (DP 6–12) and a slightly lower number of long amylopectin chains (DP 13–30) in Kinunohada than in ss2a^L^ wx (Figure 5b). This is likely because of the temperature during seed development, which was affected by the heading dates; the later the heading date, the lower the seed development temperature and gelatinization temperature [32]. SS2a wx, ss2a^L^ wx, and ss2a wx flowered in early August, similar to Akita 63, while Kinunohada is known to flower in mid-August [45], which explains the difference in peak gelatinization temperature. Therefore, considering the early flowering time of ss2a wx, its starch displayed a low gelatinization temperature. Delaying flowering time might further decrease the gelatinization temperature of ss2a wx, but this might lead to lower yield.

**Figure 6:**
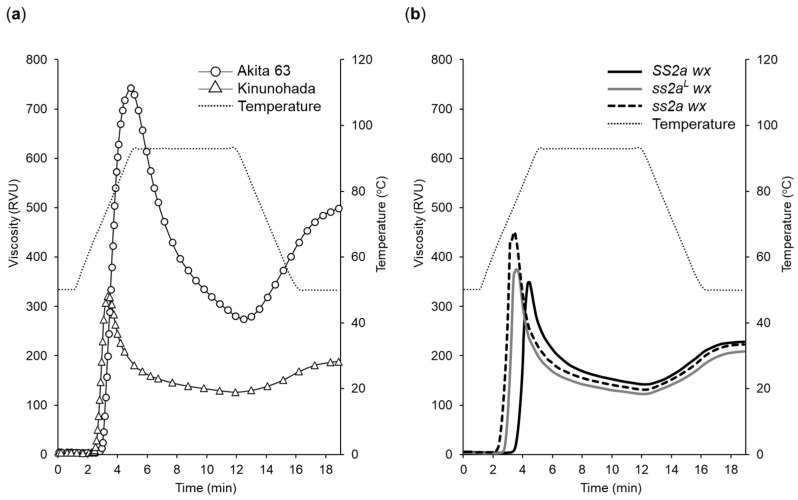
Pasting properties of endosperm starch suspension in water, as analyzed with an rapid visco analyzer (RVA). (**a**) Viscogram of Akita 63 and Kinunohada. (**b**) Comparison of the viscograms of SS2a wx, ss2a^L^ wx, and ss2a wx. Data represent the average of three replicates. Parameters of pasting properties are shown in Figure S4a. Comparison of the viscogram of Kinunohada with that of near isogenic rice lines (NILs) is shown in Figure S4b.

The pasting properties of purified starch were determined using a rapid visco analyzer (RVA). The viscograms of Akita 63, SS2a wx, ss2a^L^ wx, and ss2a wx are shown in Figure 6, and the pasting temperature, peak temperature, peak viscosity, minimum viscosity, final viscosity, breakdown, and setback of these lines are summarized in Table S1. The pasting properties of the non-glutinous rice line Akita 63 (Figure 6a) differed considerably from those of glutinous rice lines (Figure 6a,b), and these properties also varied among the glutinous rice lines with different SSIIa alleles (Figure 6, Table S1).

Pasting temperature of RVA analyses showed a strong correlation with gelatinization temperature analyzed by DSC (Table 3 and Table S1). The ss2a wx line showed the lowest pasting temperature (62.5 °C), while SS2a wx showed the highest pasting temperature (75.2 °C). The peak temperature of Akita 63 (91.3 °C) was higher than that of glutinous rice lines. Among the NILs, the peak temperature of ss2a wx was the lowest (75.8 °C), and that of SS2a wx was the highest (86.1 °C). Although the peak viscosity of Akita 63 (742 RVU) was considerably higher than that of glutinous rice lines (355–451 RVU), ss2a wx and SS2a wx showed the highest and lowest peak viscosities (451 and 355 RVU), respectively, among the NILs. These results suggest that the lower the proportion of long amylopectin chains, the higher the peak viscosity among the NILs. The final viscosity, breakdown, and setback of Akita 63 (501, 468, and 232 RVU, respectively) were also higher than those of glutinous rice lines. Although final viscosity was similar among the NILs, the breakdown and setback in SS2a wx (320 and 92 RVU, respectively) were slightly higher than those in ss2a wx (214 and 87 RVU, respectively). Comparison of the viscogram of NILs with that of Kinunohada showed that Kinunohada had the lowest peak viscosity, final viscosity, breakdown, and setback (Figure S4, Table S1).

The appearance of RVA samples incubated at room temperature for 1 h after the analyses was also different between non-glutinous and glutinous rice lines. Akita 63 turned into a white gel, while all the glutinous rice lines remained as a translucent slurry. These results show that pasting properties are affected by both amylose content and amylopectin structure.

### 2.7. Viscoelasticity of Rice Cakes

**Figure 7:**
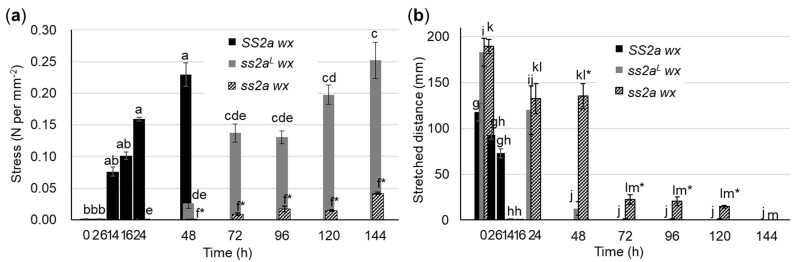
Viscoelasticity of rice cakes prepared from purified starch and stored at 4 °C. (**a**,**b**) Compression test (**a**) and stretching test (**b**) of rice lines performed using a tabletop universal tensile testing machine. Rice cakes of SS2a wx were stored at 4 °C for 0, 2, 6, 14, 16, 24, and 48 h, while those of ss2a^L^ wx and ss2a wx were stored at 4 °C for 0, 24, 48, 72, 92, 120, and 144 h. Data represent the mean ± SE of three replicates. Different lowercase letters indicate significant differences among different storage time of individual line (Tukey–Kramer method, *p* < 0.05); SS2a wx (a, b), ss2a^L^ wx (c, d, e) and ss2a wx (f) for compression tests, and SS2a wx (g, h), ss2a^L^ wx (i, j) and ss2a wx (k, l, m) for stretching tests. Asterisks indicate significant differences between ss2a^L^ wx and ss2a wx at the same storage time (t-test, *p* < 0.05).

The chain length distribution of amylopectin and the gelatinization temperature of starch are known to affect the viscoelasticity and retrogradation properties of starch [28]. The rheological properties and viscoelasticity of rice cakes are important factors determining the application of glutinous rice [46]. To reveal the effect of SSIIa alleles on viscoelasticity, rice cakes were prepared from purified starch and stored at 4 °C for different durations (0, 2, 6, 14, 16, 24, and 48 h for SS2a wx; 0, 24, 48, 72, 96, 120, and 144 h for ss2a^L^ wx and ss2a wx). Compression and stretching tests were performed using a tabletop universal tensile testing machine (EZ-test, Shimadzu) to measure the softness and stretchiness of rice cakes (Figure 7). Three replicates of rice cakes were tested in one set of experiments, and the average values of three sets of experiments performed on separate days were calculated. The results of compression and stretching tests of rice cakes showed remarkable differences among SS2a wx, ss2a^L^ wx, and ss2a wx.

Compression tests showed that the freshly prepared rice cakes of three rice lines (SS2a wx, ss2a^L^ wx, and ss2a wx) were soft (<0.001 N mm^−2^) (Figure 7a). When stored at 4 °C, the rice cake of SS2a wx hardened most rapidly compared with those of ss2a^L^ wx and ss2a wx, and its firmness increased drastically after continued cold storage (0.075 N mm^−2^ at 14 h, 0.16 N mm^−2^ at 24 h, and 0.22 N mm^−2^ at 48 h) (Figure 7a), exceeding the maximum detection limit of the machine (0.5 N mm^−2^) at 72 h, and could not be measured. By contrast, the rice cake of ss2a^L^ wx hardened slowly upon storage at 4 °C, and its firmness increased to 0.14 N mm^−2^ at 72 h and to 0.25 N mm^−2^ at 144 h (Figure 7a). Surprisingly, the rice cake of ss2a wx maintained its softness for the longest period of time at 4 °C; its firmness started to increase slightly and reached only up to 0.04 N mm^−2^ at 144 h (Figure 7a).

Stretching tests showed that the fresh rice cakes of ss2a^L^ w x and ss2a wx stretched to approximately 190 mm, while those of SS2a wx stretched less (approximately 120 mm). Upon cooling at 4 °C, the SS2a wx rice cakes showed a drastic reduction in stretchiness and completely lost their ability to stretch after 14 h of cold storage (Figure 7b). The stretchiness of ss2a^L^ wx rice cakes was maintained at 130 mm for up to 24 h of cold storage, but sharply decreased after 48 h of cold storage (Figure 7b). Remarkably, ss2a wx rice cakes maintained their stretchiness for the longest period of time, stretching to 140 mm at 48 h and even 15 mm at 120 h of cold storage.

These results suggest that SS2a wx, ss2a^L^ wx, and ss2a wx are suitable for preparing different food products. For example, SS2a wx is the most suitable for making rice crackers as it hardens rapidly, thereby minimizing preparation time. On the other hand, ss2a wx is suitable for making dumplings and rice cakes as it maintains its softness and stretchiness at cold temperatures for a long period of time.

### 2.8. Panel Evaluation of the Eating Quality of Rice Cakes

Since the pasting properties of starch and viscoelasticity of rice cakes differed considerably among SS2a wx, ss2a^L^ wx, and ss2a wx, sensory evaluation was performed to see whether these properties affect the eating quality of freshly prepared rice cakes and that of the rice cake cooked in soup after storage. Seven or more panelists aged 22–60 years participated in the present study. To evaluate fresh rice cakes, the rice cake dough was divided into equal portions, shaped into balls, and consumed within 1 h of preparation. To evaluate the rice cake cooked in soup, the chilled solid rice cake dough was sliced, cooked, and served in hot soup.

**Figure 8:**
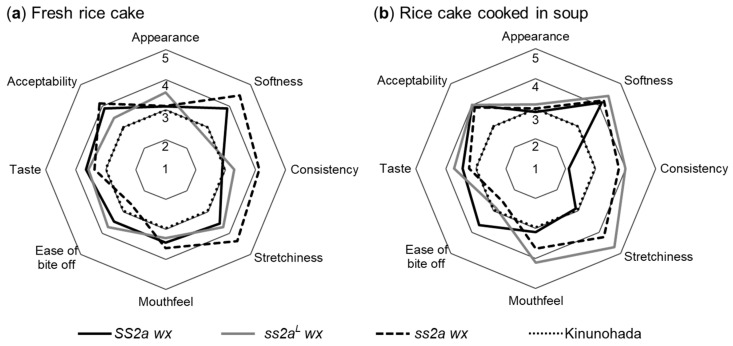
Sensory evaluation of rice cakes by panelists. (**a**,**b**) Evaluation of fresh rice cakes (**a**) and rice cakes cooked in soup (**b**) on a 5-point hedonic scale by at least seven panelists. Data represent mean of at least seven panelists. The commonly consumed glutinous rice cultivar Kinunohada was used as a control, and its score was set at three.

Pairwise comparisons of the three NILs (SS2a wx, ss2a^L^ wx, and ss2a wx) were performed against the commonly consumed glutinous rice cultivar Kinunohada (control) in the Akita region. Evaluation was performed using a five-point hedonic scale, and scores for Kinunohada were set at three. Sensory parameters including appearance, softness, consistency, stretchiness, mouthfeel, ease of bite off, taste, and acceptability were evaluated (Figure 8). Fresh rice cakes prepared from ss2a wx were the softest and showed the highest consistency and stretching ability but were more difficult to bite off compared with those prepared from SS2a wx, ss2a^L^ wx, and Kinuohada. Fresh rice cakes prepared from SS2a wx, ss2a^L^ wx, and ss2a wx showed similar appearance, mouthfeel, and taste, and their acceptability was higher than those prepared from Kinunohada. Rice cakes cooked in soup exhibited similar softness, taste, and acceptability among SS2a wx, ss2a^L^ wx, and ss2a wx. The softness of rice cakes varied with the cooking method, probably because rice cakes served in the hot soup did not retrograde unlike fresh rice cakes, which were served at room temperature and likely retrograded to some degree. The rice cakes of SS2a wx served in soup showed lower consistency and stretchiness but were easier to bite off than those of ss2a^L^ wx and ss2a wx; this result was in contrast with the evaluation of fresh rice cakes. The consistency of rice cakes and the ease of bite off showed an inverse relationship for both fresh and cooked rice cakes; the less sticky the rice cake, the easier the process of chewing and swallowing. Thus, SS2a wx would be the most suitable NIL for preparing rice cakes to be cooked in soup and served to the elderly and young children, while ss2a wx would be most suitable for preparing rice cakes to be served fresh. The taste and acceptability of SS2a wx, ss2a^L^ wx, and ss2a wx rice cakes scored higher than those of Kinunohada rice cakes, suggesting that these three NILs are suitable for commercial use, and that some other factors such as proteins, lipids, and other aromatic compounds potentially influence the taste of rice cakes.

## 3. Discussion

### 3.1. Effect of SSIIa Alleles on Starch Structure and Its Relationship to Rice Thermal Properties, Viscoelasticity, and Eating Quality

Numerous glutinous rice lines have been utilized for different purposes to date, depending on their characteristics [23]. However, how those characteristics are determined remains unclear. The present study revealed the precise effects of SSIIa on the various properties of glutinous rice using NILs. The use of NILs allowed us to eliminate any possible effects of mixed genetic backgrounds. For example, among genotypes with different heading dates, the structure of starch is affected to some extent by changes in environmental conditions, such as temperature, during seed development [32]. Glutinous rice NILs with high, low, or no SSIIa activity (designated as *SS2a wx*, *ss2a^L^ wx*, and *ss2a wx*, respectively) were generated by backcrossing using the large seeded, high yielding, early flowering, elite japonica rice cultivar Akita 63 as a recurrent parent (Figure 1, Figure 2 and Figure 3, Table 1).

The presence of the *wx* allele derived from EM21 in *SS2a wx*, *ss2a^L^ wx*, and *ss2a wx* was determined using the dCAPS marker, and the mutation site in these NILs was confirmed to be different from that in Kinunohada (Figure 1d). Absence of GBSSI in *SS2a wx*, *ss2a^L^ wx*, *ss2a wx*, and Kinunohada was confirmed by Western blotting (Figure 2). The seeds of these rice NILs displayed a white opaque phenotype upon desiccation (Figure 4), indicative of glutinous rice [47]. Iodine staining of rice seed cross-sections (Figure 4) and gel filtration analyses of purified starch (Table 2, Figure S2) showed that three NILs and Kinunohada contained essentially no amylose. This confirmed that the NILs generated in the present study were glutinous. The genotype of *SSIIa* in NILs was determined using a dCAPS marker (Figure 1a–c). The amount of SSIIa in the total protein extract was higher in *SS2a wx* than in *ss2a^L^ wx* and was absent in *ss2a wx.* SSIIa was strongly associated with starch granules in *SS2a wx* but not in *ss2a^L^ wx* and *ss2a wx* (Figure 2). This is in agreement with the results of a previous study, which showed that SSIIa is strongly associated with starch granule proteins in rice accessions harboring the *SS2a* allele [3]. Additionally, SSIIa activity was observed in *SS2a wx*, although its signal was faint (Figure 3).

Chain length distribution analyses of amylopectin showed that low SSIIa activity corresponds to a high proportion of short amylopectin chains (DP < 12) (Figure 5). The chain length distribution of amylopectin showed a tight correlation with the thermal properties, such as peak gelatinization temperature (Table 3), pasting temperature (Table S1), and viscoelasticity of the rice cakes (Figure 7). The number of short amylopectin chains (DP < 12) was higher, and the gelatinization and pasting temperatures were lower in *ss2a wx* than in *SS2a wx* and *ss2a^L^ wx* (Figure 5 and Figure 6, Table 3 and Table S1. This is consistent with the results of previous studies on non-glutinous rice with different *SSIIa* genotypes [20,21,34].

The effect of amylopectin branch structure on the gelatinization temperature and pasting properties of glutinous rice is also supported by the loss of other starch biosynthetic enzymes or substitutions in their amino acid sequence. For example, the loss of BEI in Aichimochi 126, derived from Hiderishirazu-D due to a transposon insertion into the 2^nd^ intron of *BEI* resulted in an increase in the number of short amylopectin chains (DP 5–12) [30,31], which lowered its pasting temperature (66 °C) by 5 °C compared with that of Himenomochi (71.3 °C), resulting in softer rice cakes [31]. By contrast, the loss of BEIIb in *wx ae*, derived from EM16 (selected from the MNU-mutagenized population of Kinmaze), decreased the number of short amylopectin chains (DP < 15) and increased the number of long amylopectin chains (DP > 15), which in turn increased the gelatinization temperature (to 83 °C) as well as the pasting temperature [48]. Similarly, Shirokumamochi, possessing BEIIb derived from Kitaake, contains Leu94Val and His196Arg substitutions and produced harder rice cakes compared to Kitayukimochi [49], likely because of a slight reduction in BEIIb activity. Although the genetic factors affecting amylopectin branch structures in the vast majority of other glutinous rice cultivars remain unknown, the results of the present study support the idea that rice lines containing starch with fewer short amylopectin chains and more long amylopectin chains exhibit higher gelatinization and pasting temperatures and produce harder rice cakes, whereas those with starch containing more short amylopectin chains and fewer long amylopectin chains exhibit lower gelatinization and pasting temperatures and produce softer rice cakes [50,51].

### 3.2. Characteristics and Applications of Rice NILs

Glutinous rice is steamed and consumed as a staple in Laos and Thailand [52], and is also used as an ingredient in a wide variety of dishes in many Asian countries. For example, in Japan, polished glutinous rice is used to make celebratory steamed dishes with red beans (*Sekihan*) and meat and/or seasonal vegetables (*Okowa*). Glutinous rice is also used for making rice cakes (*Mochi*), confectioneries (*Daifuku)*, rice crackers (*Okaki* and *Arare*), and dumplings (*Dango*). In addition, glutinous rice is used for brewing cooking liquors, such as Japanese Mirin and Chinese Shaoxing wine, and for producing vinegar by fermenting. The requirements of starch, other than being amylose-free, depend on the end products.

The present study revealed that the genotype of *SSIIa* in rice greatly affects the structure of amylopectin, which in turn affects the physical properties (Figure 6 and Figure 7) and sensory parameters (Figure 8) of *SS2a wx*, *ss2a^L^ wx*, and *ss2a wx*. Rice cakes made from *ss2a wx* were considerably softer and stretchier than those made from *ss2a^L^ wx*, and even softer and stretchier than those prepared from *SS2a wx* (Figure 7). The mechanical testing parameters of rice cakes made from purified starch correlated well with some sensory evaluation parameters of rice cakes made from polished grains (Figure 7 and Figure 8). Particularly, the consistency and stretchiness of fresh rice cakes showed a close relationship with the *SSIIa* allele, and the rice cakes made from *ss2a wx* showed the highest consistency and stretchiness (Figure 8). The softness of rice cakes served in soup was similar among the three NILs but different for Kinunohada. This suggests that other properties, such as starch macrostructure and protein and/or fiber content, affect the characteristics of rice cakes served in hot soup.

When glutinous rice is used for making sliced rice cakes or rice crackers, the dough should not be too sticky and should preferably firm up rapidly to minimize processing time. Therefore, *SS2a wx* is suitable for making sliced rice cakes and rice crackers. The *ss2a wx* line is suitable for making rice cakes (*Mochi*) and confectioneries (*Daifuku)*, and their quality is maintained for a longer period of time, even at lower temperatures, reducing food loss and waste. In addition, rice cultivars with more short amylopectin chains produce higher alcohol content and are used for brewing Sake [53]. In fact, use of Aichi 126 for Mirin brewing increased its yield [31]. Therefore, *ss2a wx* could be used to increase Mirin yield and decrease waste. Further evaluation of the production and sensory parameters of different food products made from NILs generated in this study compared with those made from pre-existing glutinous rice cultivars will allow identification of their best application. Although *ss2a^L^ wx* has similar starch properties to many other pre-existing glutinous rice cultivars, all three NILs generated in this study have a large grain size, which is a desirable milling trait for producing polished rice grains.

### 3.3. Characteristics of NILs and Their Potential as New Rice Cultivars

Genotyping revealed that the mutation site in the *wx* allele of NILs, derived from EM21, was different from that in the *wx* allele of Kinunohada (Figure 1d). The *wx* allele of Kinunohada was derived from Chubumochi 37, which likely carried the *wx* allele from Nijiginmochi or Iwaimochi, ancestral Japanese glutinous rice cultivars [45], although its causal mutation site(s) in *Waxy* gene are currently unknown. At least 26 known haplotypes of the *wx* allele have been reported in glutinous rice lines grown worldwide. In addition, nucleotide polymorphisms have been observed in other starch biosynthetic genes, such as *SSI*, *SSIIa*, and/or *SSG6* (which encodes an aminotransferase), in glutinous rice lines grown in Southeast and East Asian countries, which underwent the domestication process [23]. The use of the *wx* haplotype derived from EM21 will add another dimension to the selection of the *wx* allele during the breeding process of glutinous rice lines by taking advantage of the dCAPS marker generated in the present study.

According to the rice cultivar database search engine governed by the Institute of Crop Science in National Agriculture and Food Research Organization (https://ineweb.narcc.affrc.go.jp/search/ine.cgi?action=tokusei_menu, accessed on 2 December 2022), a total of 1188 registered glutinous rice lines are currently registered in Japan, although numerous nonregistered rice lines also exist. Among the 339 cultivars registered with detailed information, the thousand grain weight was <20 g for 80 cultivars, 20–22 g for 170 cultivars, 22–24 g for 79 cultivars, undisclosed for several cultivars, and >25 g for only two cultivars (Miyatamamochi, 29.6–31.0 g; Yashiromochi, 25.6 g). However, both Miyatamamochi and Yashiromochi grow in low latitude areas with warm climates, and flower late (at the end of August). Therefore, both these cultivars cannot be cultivated in high latitude areas, since winter arrives early, and temperatures sharply decline in early September in these regions. Thus, it is important to select rice lines with the early flowering trait to ensure optimum temperature during seed development and to maximize yield. Although detailed surveys of agricultural traits are required, our preliminary observation of the flowering time of NILs was in early August (August 2–3, 2021), which showed that their thousand grain weight was 27 g (Table 2). This implies that the NILs generated in the present study could be cultivated in high latitude areas.

## 4. Materials and Methods

### 4.1. Plant Materials

To develop *SS2a wx* and *ss2a^L^ wx* NILs, the non-glutinous indica rice cultivar IR36 (*SS2a Wx^a^*) was crossed with a japonica glutinous rice mutant, EM21 (*ss2a^L^ wx* [10,11]). The original *SS2a wx* genotype was isolated from the F_2_ population and backcrossed three times with an early flowering, large seeded, high yielding elite japonica rice cultivar, Akita 63 (*ss2a^L^ Wx^b^* [37]). Finally, NILs with the *SS2a wx* and *ss2a^L^ wx* genotypes were isolated from the BC_3_F_2_ population.

To generate the *ss2a wx* NIL, the non-glutinous *ss2a* japonica rice mutant EM204 (*ss2a Wx^b^* [21]), which was previously isolated from the MNU-mutagenized population of the wild-type japonica cultivar Kinmaze [21], was backcrossed three times with Akita 63 and then crossed with *ss2a^L^ wx* NILs. Finally, NILs with the ss*2a wx* genotype were isolated.

The BC_3_F_5_ or BC_3_F_6_ generations of NILs were used for analyses in the present study. Theoretically, at least 93.75% of the genome of each NIL was derived from Akita 63. All rice lines were grown in an experimental paddy field in Akita Prefecture (Japan) during summer under natural light conditions, except Kinunohada, which was grown in Akita Prefecture but purchased from a local grocery store.

### 4.2. Genotyping of SSIIa and Wx

The *SSIIa* gene was genotyped as described previously [21,37]. To genotype the *wx* allele derived from EM21, a PCR was performed using the Quick Taq HS dye mix (TOYOBO, Osaka, Japan) and sequence-specific primers (5′-GGAACTTATGGTGAGTTACAATTGATCTCAAG-3′ and 5′-GTTCTTCAGGTAGCTCGTCAGTGGGTCAG-3′) under the following conditions: 94 °C for 2 min, and 38 cycles of 94 °C for 20 s, 60 °C for 20 s, and 68 °C for 20 s. PCR products were digested by *Bse*NI at 60 °C and separated by electrophoresis on 7.5% acrylamide gel in 1X TBE buffer. The expected sizes of the PCR products were 167 bp for the *wx* mutant and 135 and 32 bp for the wild type.

### 4.3. Western Blot Analysis of SSI, SSIIa, and GBSSI

Total protein and starch granule-bound protein (both loosely and tightly bound protein) were extracted from one mature seed of each genotype, and Western blotting was performed as described previously [3].

### 4.4. Zymogram Analyses of Starch Biosynthetic Enzymes

Soluble protein was extracted from seeds at the mid-developmental stage, and zymogram analyses were performed as described previously [35].

### 4.5. Amylose Content and Amylopectin Structure Analyses

Starch was purified using the cold-alkaline method, as described previously [54,55]. To determine the apparent amylose content, the purified starch was debranched using *Pseudomonas* isoamylase (Hayashibara, Okayama, Japan) and analyzed by gel filtration chromatography (Toyopearl HW-55S and HW-50S×3, LC-8020 Model II software version 4.01; Tosoh, Tokyo, Japan) [56,57,58]).

To determine the amylopectin structure, debranched purified starch was fluorescently labeled and analyzed by capillary electrophoresis (32 Karat software version 10.2, P/ACE MDQ Plus Carbohydrate System; AB Sciex, Framingham, MA, USA), as described previously [59].

### 4.6. Thermal and Pasting Properties of Starch

The thermal properties of purified starch were analyzed by DSC (LabSolutions TA software version 1.01, DSC 60 Plus; Shimadzu, Kyoto, Japan). Briefly, 3 mg of purified starch was suspended in 9 μL of distilled water and encapsulated in an aluminum seal pan (S201-53090; Shimadzu, Kyoto, Japan). Then, 45 mg of activated alumina was encapsulated in the aluminum seal pan and used as a reference sample. The thermal properties of starch were analyzed using the temperature program described previously [60,61].

The pasting properties of purified starch were analyzed by RVA (Thermoclin software version 2.1, RVA-4; Newport Scientific, Inc., Jessup, MD, USA) using 3.5 g of purified starch suspended in 25 mL of distilled water, as described previously [60].

### 4.7. Physical Properties of Rice Cakes

#### 4.7.1. Rice Cake Preparation

Purified starch was mixed with 1.5 volumes (*w*/*v*) of distilled water. The mixture was autoclaved, poured into a container (40 mm diameter, 12 mm depth), and covered with a lid. Rice cakes were stored at 4 °C until evaluation.

#### 4.7.2. Compression Test

Rice cake in a container was pressed downward with a cylindrical press jig (20 mm diameter) from the surface of the rice cake to 6 mm at a test speed of 60 mm min^−1^, and the stress was measured using a tabletop universal tensile tester (Trapezium X software version 1.5.3, EZ-test; Shimadzu, Kyoto, Japan).

#### 4.7.3. Stretching Test

After compressing the rice cake, the jig was pulled upward at a test speed of 60 mm min^−1^ until the rice cake dough snapped, and the distance was recorded.

### 4.8. Sensory Evaluation of Rice Cakes

#### 4.8.1. Rice Cake Preparation

Two cups of polished rice were washed with water and then soaked in water for 16 h at 4 °C. Completely drained rice was steamed with 320 mL of water for 25 min and pounded for 15 min using a rice cake dough maker (Mochikko, PFC-20FK; Toshiba, Tokyo, Japan). To test fresh rice cakes, the dough was shaped into balls (3 cm diameter) using a rice cake dough dispenser (Marumochi-kun, SMX-5401; Tiger corporation, Osaka, Japan). To test rice cakes served in soup, the dough was rolled out into 1.5 cm thickness, chilled for 2 days at 4 °C, sliced into 3 cm × 2 cm sections, and stored at *−*30 °C until use. Frozen rice cakes were returned to room temperature and cooked in a soup (8-fold-diluted Ajidouraku; Tohoku Soy Sauce Corporation, Akita, Japan) for 5 min.

#### 4.8.2. Sensory Evaluation

Seven to nine panelists (four to six females and three males; 22–63 years of age) participated in the paired comparison tests. Evaluation was performed using a 5-point hedonic scale, and scores for Kinunohada were set at 3. Sensory parameters included appearance, softness, consistency, stretchiness, mouthfeel, ease of bite off, taste, and acceptability. Results were expressed as the mean of triplicate analyses.

## 5. Conclusions

This study evaluated the precise effect of SSIIa activity on glutinous rice properties. NILs were used in this study to eliminate the effect of other factors that could alter starch structure, such as flowering time, temperature during seed development, and the presence of SNPs in other starch biosynthetic enzymes. Although SSIIa from indica rice shows higher activity than that from japonica rice, which affects the properties of glutinous starch, how the complete loss of SSIIa affects the structure of amylopectin, thermal and pasting properties of starch, and viscoelasticity and sensory parameters of rice cakes was unknown.

Comparison among *SS2a wx*, *ss2a^L^ wx*, and *ss2a wx* clearly showed that the lower the SSIIa activity, the higher the number of short amylopectin chains (DP 6–12). Variation in chain length distribution pattern directly influenced the gelatinization temperature and pasting properties of starch; the greater the number of short amylopectin chains, the lower the gelatinization temperature and peak viscosity of starch. This affected the retrogradation properties of starch. Lowering the activity of SSIIa maintained the softness of rice cakes for a longer period of time and increased the stretching ability of rice cakes.

These results suggest that *SS2a wx* is the most suitable NIL for preparing rice crackers as it firms up quickly, thus minimizing preparation time. The *ss2a^L^ wx* NIL would be suitable for preparing steamed rice dishes because it is neither too soft nor too hard. By contrast, *ss2a wx* is suitable for preparing dumplings and sweets (as it maintains softness at cold temperature), as well as for brewing Mirin, a glutinous rice wine. Taken together, the *SS2a wx*, *ss2a^L^ wx*, and *ss2a wx* NILs generated in this study provided detailed information for controlling the properties of glutinous rice. These NILs could serve as new genetic materials for breeding novel glutinous rice cultivars and could be utilized as ingredients in various food products.

## Figures and Tables

**Table 1:** Summary of *SSIIa* and *Wx* genotypes in rice lines.

Rice Line	*SSIIa* Allele ^1^	*Wx* Allele ^2^
Akita 63	*ss2a^L^*	*Wx^b^*
*SS2a wx*	*SS2a*	*wx*
*ss2a^L^ wx*	*ss2a^L^*	*wx*
*ss2a wx*	*ss2a*	*wx*
IR36	*SS2a*	*Wx^a^*
EM21	*ss2a^L^*	*wx*
EM204	*ss2a*	*Wx^b^*
Kinunohada ^3^	*ss2a^L^*	*wx*

^1^ Superscript L denotes the leaky mutation present in the SSIIa allele of wild-type japonica rice. ^2^ Wx^a^ allele exists in typical indica rice, and Wx^b^ allele exists in typical japonica rice accessions. Glutinous rice lines possess the wx allele. ^3^ The wx allele in Kinunohada is different from that in SS2a wx, ss2a^L^ wx, ss2a wx, and EM21.

**Table 2:** Seed weight and amylose content.

Rice Line	Average Seed Weight ^1^ (mg grain^−1^)	Apparent Amylose Content ^2^ (%)
Akita 63	31.7 ± 0.5a	17.1 ± 0.5a
*SS2a wx*	27.8 ± 0.5b	0.5 ± 0.2b
*ss2a^L^ wx*	27.1 ± 0.4b	1.0 ± 0.4b
*ss2a wx*	27.5 ± 0.5b	2.1 ± 0.1b
EM21	17.6 ± 0.2c	0.5 ± 0.1b ^3^
Kinunohada	23.6 ± 0.3d	1.0 ± 0.1b

^1^ Data represent the mean ± standard error (SE) of 20 dehulled grains. ^2^ Apparent amylose content corresponding to fraction I of the gel filtration chromatography elution profile was calculated (Figure S2). Data represent the mean ± SE of three replicates. Different lowercase letters indicate significant differences (Tukey–Kramer method; *p* < 0.05). ^3^ Previously reported data [3].

**Table 3:** Gelatinization temperature of purified endosperm starch determined by DSC.

Rice Line	T_o_ (°C) ^1^	T_p_ (°C) ^1^	T_c_ (°C) ^1^	DH (J/g) ^1^
Akita 63	56.6 ± 0.1b	63.0 ± 0.0c	70.6 ± 0.1c	13.3 ± 0.6b
*SS2a wx*	67.0 ± 0.2a	73.0 ± 0.2a	82.9 ± 0.7a	19.2 ± 0.4a
*ss2a^L^ wx*	57.8 ± 0.1b	64.7 ± 0.1b	74.7 ± 0.1b	17.1 ± 0.8ab
*ss2a wx*	50.0 ±0.1d	60.7 ±0.3d	72.0 ±0.2c	14.1 ± 0.5b
Kinunohada	54.0 ± 0.2c	61.6 ±0.1d	70.6 ±0.1c	15.4 ± 0.2ab

^1^ Data represent the mean ± SE of three replicates. Different lowercase letters indicate significant differences (Tukey–Kramer method; *p* < 0.05). T_o_, Onset temperature; T_p_, peak gelatinization temperature; T_c_, conclusion temperature; ΔH, gelatinization enthalpy.

## Data Availability

Not applicable.

## Supplementary Materials

The supporting information can be downloaded at: https://www.mdpi.com/article/10.3390/ijms24043726/s1.
