# Supplementary material for: Three Starch Synthase IIa (SSIIa) Alleles Reveal the Effect of SSIIa on the Thermal and Rheological Properties, Viscoelasticity, and Eating Quality of Glutinous Rice
Source: Int J Mol Sci. 2023 Feb 13;24(4):3726. doi: 10.3390/ijms24043726 (PMC9962474; doi:10.3390/ijms24043726)
Supplement: Supplementary file 1 [file ijms-24-03726-s001.zip › ijms-2198358-supplementary.pdf]

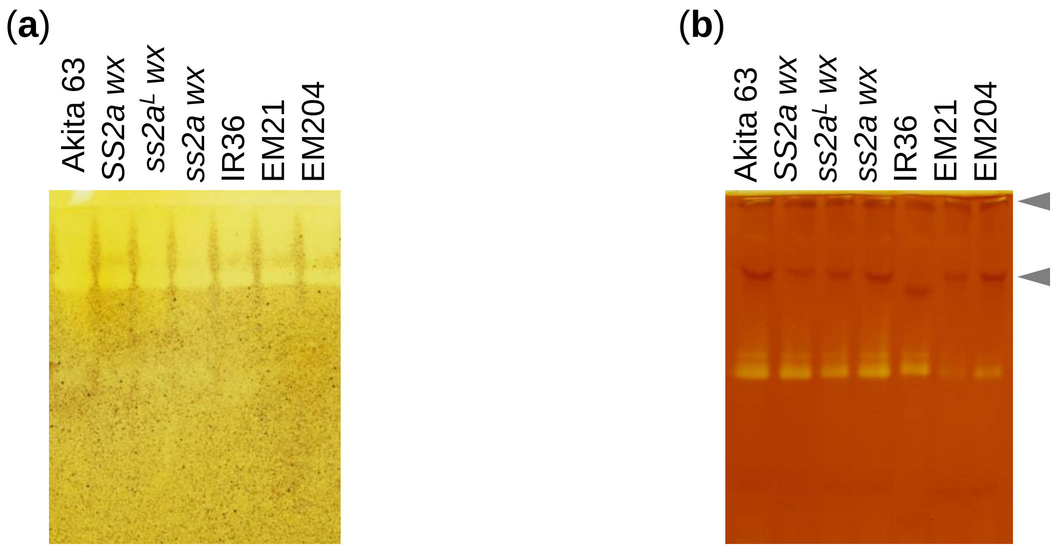

Figure S1. Control gels for zymograms. Reactions were performed in the absence of substrate, ADP-glucose. (**a**) Control gel for starch synthase (SS) IIa activity. (**b**) control gel for SSI and SSIIIa activities. Gray arrow heads are likely transferase or hydrolase activities, and removal of short glucan branches by these enzymes probably generated linear chains which can bind to iodine.

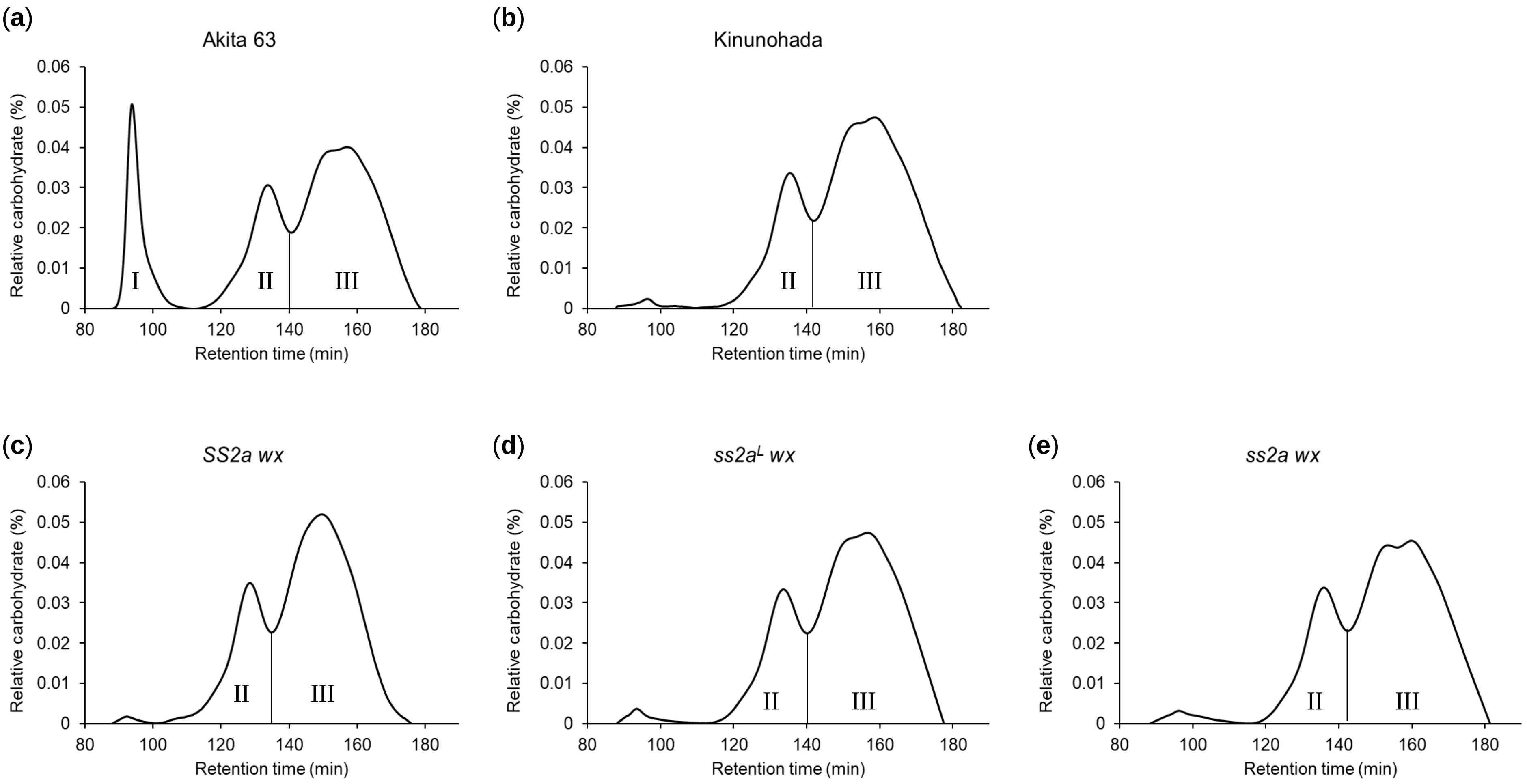

Figure S2. Profiles of gel filtration chromatography. Debranched starch prepared from rice seeds were separated by a series of single HW-55S and triple HW-50S Toyopearl columns. Three fractions (I, II, and III) were divided at the troughs of carbohydrate-content curve. Amylose elutes in fraction I, long chains of amylopectin branches elute in fraction II, and short chains of amylopectin branches elute in fraction III. Elution profiles are one of the representatives of at least three experiments. Exact values are shown in Table 2.

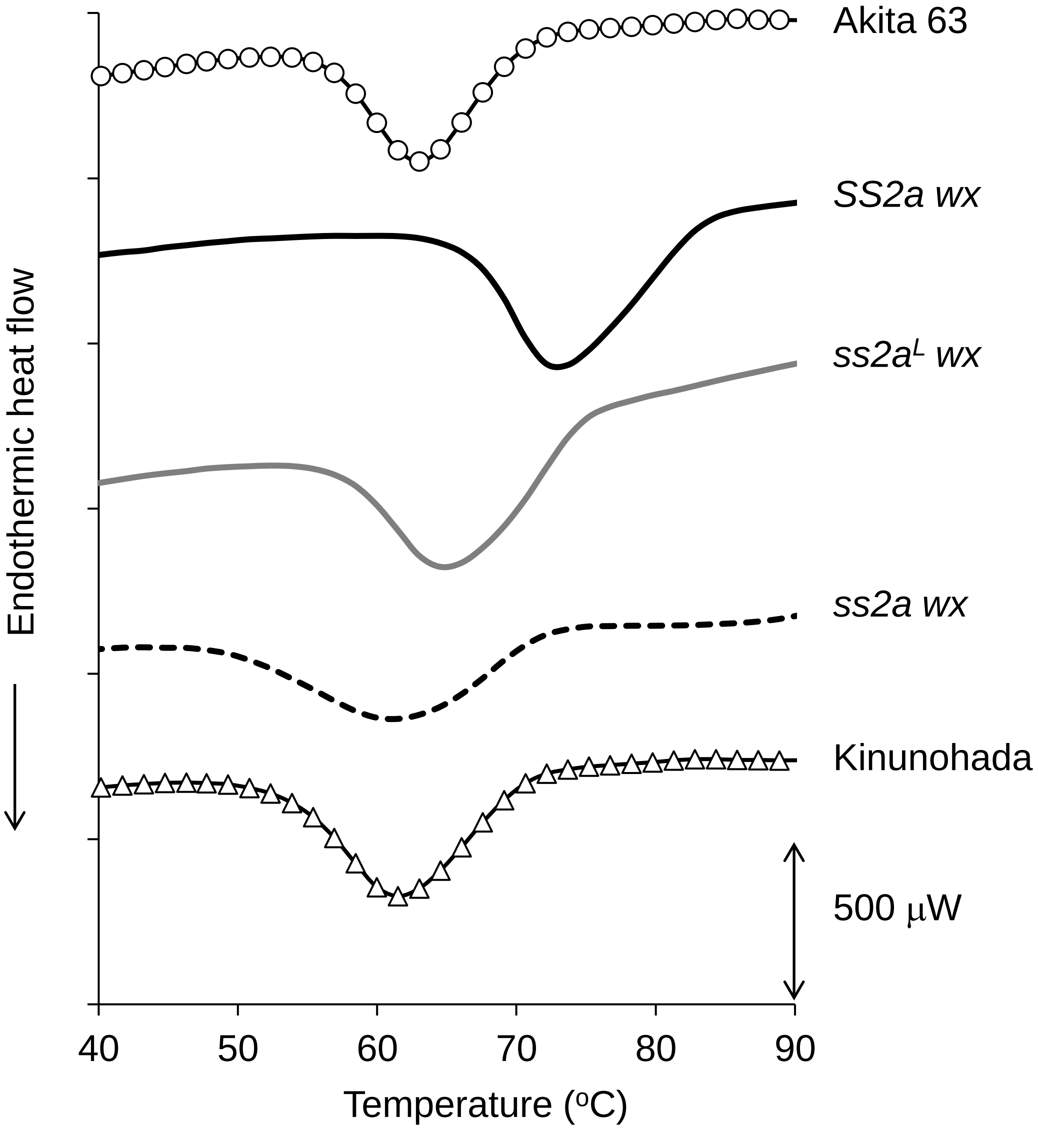

Figure. S3 : Profiles of differential scanning calorimetry using purified starch from rice seeds. One of the representatives data sets of at least three experiments is shown. Exact values of onset, peak gelatinization, and conclusion temperatures and gelatinization enthalpy are summarized in Table 3.

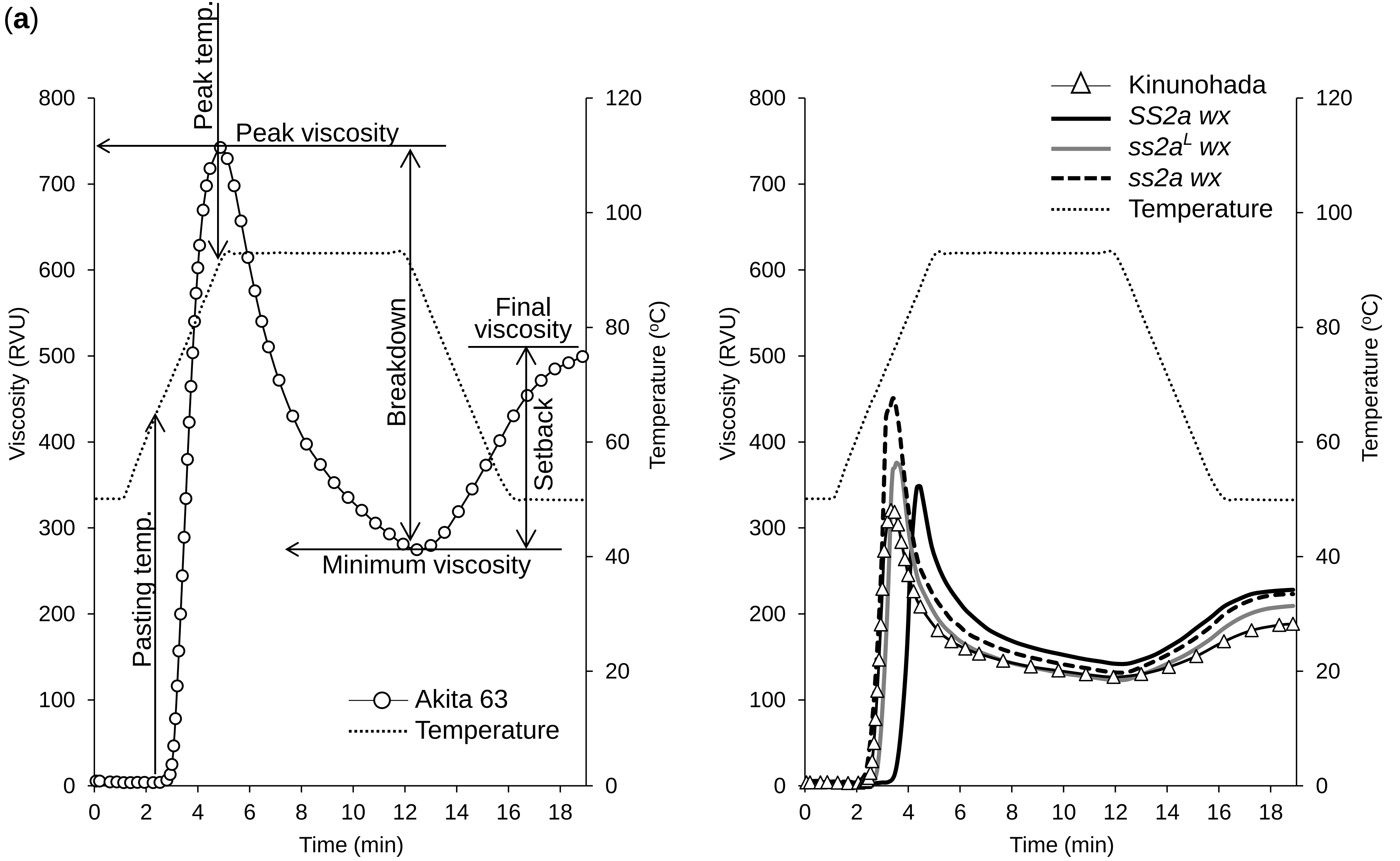

Figure. S4 : Parameters and profiles of viscogram. (**a**) Parameters of viscogram are indicated in the figure, which include pasting temperature, peak temperature, minimum viscosity, peak viscosity, final viscosity, breakdown, and setback. (**b**) Comparison of viscogram among glutinous rice lines including Kinunohada.

Table S1. Parameters of pasting properties analyzed by rapid visco analyzer (RVA)

Rice line	Pasting temperature (°C)	Peak temperature (°C)	Peak Viscosity (RVU)	Minimum viscosity (RVU)	Final Viscosity (RVU)	Breakdown (RVU)	Setback (RVU)
Akita 63	69.8 ± 0.2b	91.3 ± 0.0a	742.4 ± 5.1a	274.0 ± 7.5a	500.5 ± 4.1a	468.3 ± 10.9a	232.4 ± 0.3a
SS2a wx	75.2 ± 0.2a	86.1 ±0.2b	354.8 ± 2.1c	140.9 ± 2.0b	227.6 ± 2.2b	214.0 ± 2.9cd	86.8 ± 0.2bc
ss2a^{L} *wx*	67.6 ± 0.3c	77.4 ± 0.5c	377.7 ± 1.8c	122.2 ± 2.0b	209.9 ± 4.1bc	255.5 ± 3.9c	87.6 ± 6.0bc
ss2a wx	62.5 ± 0.3e	75.8 ± 0.2c	451.0 ± 4.0b	130.8 ± 3.1b	223.2 ± 2.6b	320.2 ± 6.6b	92.4 ± 5.6b
Kinunohada	65.0 ± 0.2d	75.3 ± 0.2c	321.9 ±2.3d	125.4 ± 0.6b	188.7 ± 0.3c	196.5 ± 2.1d	63.3 ± 0.6c

Data represent means ± SE of three replicates. Different lowercase letters indicate significant differences by Tukey-Kramer method ($p < 0.05$).
